# Insulation of a synthetic hydrogen metabolism circuit in bacteria

**DOI:** 10.1186/1754-1611-4-3

**Published:** 2010-02-25

**Authors:** Christina M Agapakis, Daniel C Ducat, Patrick M Boyle, Edwin H Wintermute, Jeffrey C Way, Pamela A Silver

**Affiliations:** 1 Department of Systems Biology, Harvard Medical School, Boston, MA 02115, USA; 2 Wyss Institute for Biologically Inspired Engineering, Harvard University, Boston, MA 02115, USA

## Abstract

**Background:**

The engineering of metabolism holds tremendous promise for the production of desirable metabolites, particularly alternative fuels and other highly reduced molecules. Engineering approaches must redirect the transfer of chemical reducing equivalents, preventing these electrons from being lost to general cellular metabolism. This is especially the case for high energy electrons stored in iron-sulfur clusters within proteins, which are readily transferred when two such clusters are brought in close proximity. Iron sulfur proteins therefore require mechanisms to ensure interaction between proper partners, analogous to many signal transduction proteins. While there has been progress in the isolation of engineered metabolic pathways in recent years, the design of insulated electron metabolism circuits *in vivo *has not been pursued.

**Results:**

Here we show that a synthetic hydrogen-producing electron transfer circuit in *Escherichia coli *can be insulated from existing cellular metabolism via multiple approaches, in many cases improving the function of the pathway. Our circuit is composed of heterologously expressed [Fe-Fe]-hydrogenase, ferredoxin, and pyruvate-ferredoxin oxidoreductase (PFOR), allowing the production of hydrogen gas to be coupled to the breakdown of glucose. We show that this synthetic pathway can be insulated through the deletion of competing reactions, rational engineering of protein interaction surfaces, direct protein fusion of interacting partners, and co-localization of pathway components on heterologous protein scaffolds.

**Conclusions:**

Through the construction and characterization of a synthetic metabolic circuit *in vivo*, we demonstrate a novel system that allows for predictable engineering of an insulated electron transfer pathway. The development of this system demonstrates working principles for the optimization of engineered pathways for alternative energy production, as well as for understanding how electron transfer between proteins is controlled.

## Background

Metabolic electron transfer is a well-characterized process [1], understood at the level of engineering principles [2]. Electrons readily tunnel between iron-sulfur clusters, whose electrical potential is defined by the chemical environment created by the amino acids that surround them in the protein matrix [3]. As with other biological systems, including signal transduction [4], complex electron transfer pathways likely evolved through gene duplication, recombination, and drift of iron-sulfur containing proteins, with interaction between two iron sulfur containing proteins or domains largely controlled by electrostatic forces [5]. Taking advantage of the modular nature of iron sulfur protein interactions, several groups have recombined redox proteins *in vitro *in order to engineer novel electron transfer pathways [6]. Much of the focus in previous work has been to create physical interfaces between electrodes and enzymes [7], joining electron transfer proteins with electron-generating proteins through "molecular Lego" *in vitro *[8].

There are many appealing applications for such engineered electron transfer systems *in vitro*, such as miniaturized biofuel cells, biocatalysts, and biosensors [7]. These approaches, however, do not take advantage of the self-assembly and self-regenerating abilities of live cells. Engineered cellular pathways *in vivo *have the potential to impact our understanding of cellular electron transfer systems in live cells and may provide self-renewing platforms for the continuous production of fuels and other useful molecules [9].

To this end, we chose to design a synthetic electron transfer circuit in *E. coli *that utilizes [FeFe]-hydrogenases, a class of metalloenzymes that catalyse the reversible reduction of protons to molecular hydrogen [10]. Previous research using hydrogenases within engineered electron transfer pathways have relied heavily on *in vitro *approaches. For example, hydrogenase enzymes have been explored as tools for hydrogen fuel production by purified enzyme cocktails *in vitro *[11]. Alternatively, the hydrogenase active site has been modeled synthetically for use in fuel cells as a catalytic center that does not require rare metals to function [12]. While these *in vitro *systems are inherently insulated from natural metabolism, they suffer from the same drawbacks as other *in vitro *enzymatic processes in the difficulty of production and purification, and lack of robustness from the living cell. Metabolic [13] and protein engineering [14] of natural hydrogen production pathways in *E. coli *have yielded improvements in hydrogen yield, but these methods are limited in the substrate specificity of the native [NiFe]-hydrogenases [15]. Synthetic pathways expressing hydrogenases along with exogenous electron donors and carriers can be used to supplement and optimize hydrogen production from *E. coli*, as well as improve our understanding of electron transfer pathways.

Natural hydrogen metabolism pathways in a variety of prokaryotic and eukaryotic species can either consume hydrogen as a source of low potential electrons, or produce hydrogen as a sink for reducing equivalents generated during anaerobic fermentation. In *E. coli*, both hydrogen consumption [16] and production [17] are performed by several [NiFe]-hydrogenases. While the native *E. coli *[NiFe]-hydrogenases are coupled to NADH, with a reducing potential of -320 mV, [FeFe]-hydrogenases are partnered with the electron carrying protein ferredoxin, which can have a significantly stronger reducing potential, typically close to that of the H_2_/H^+ ^pair (-420 mV) [10]. As a result, [FeFe]-hydrogenases thermodynamically favor hydrogen production. Moreover, their high hydrogen production activity, conserved structure, and relatively simple maturation pathway make [FeFe]-hydrogenases excellent enzymatic modules for recombinant expression in a synthetic system. Furthermore, heterologously expressed [FeFe]-hydrogenases from several species have been characterized *in vitro *[18] and *in vivo *[19]

**Figure 1:**
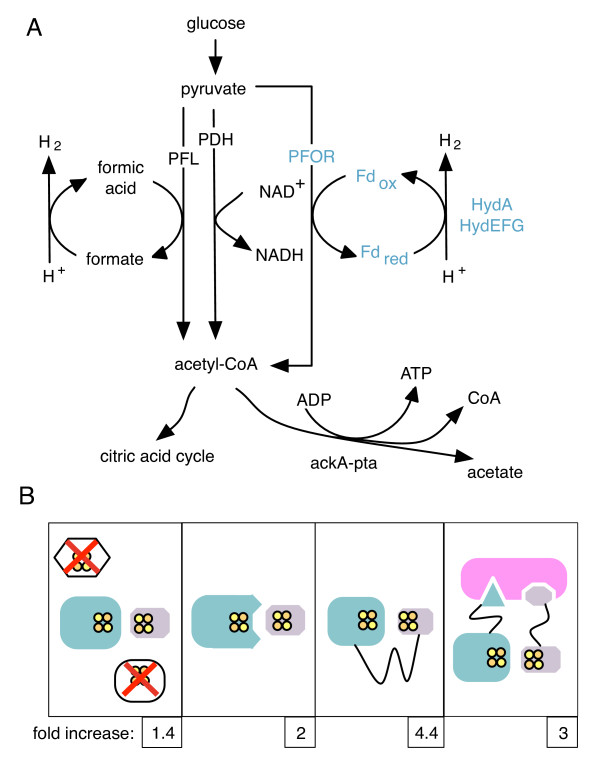
**Overview of synthetic pathway design and insulation strategies **A.) Natural and synthetic pyruvate metabolism to acetyl-CoA in *E. coli *through the pyruvate dehydrogenase complex (PDH), pyruvate formate lyase (PFL), and the heterologous PFOR-ferredoxin (Fd)-hydrogenase synthetic pathway. Native enzymes are indicated in black, heterologous enzymes in blue.~~~B.) Insulation strategies for synthetic electron transfer pathways; deletion of competing reactions, optimization of binding surfaces, direct protein-protein fusion, and localization to a synthetic protein scaffold. We present the maximum fold increase in hydrogen production due to each method, calculated by comparing normalized values of hydrogen production by otherwise identical synthetic pathways with and without the insulation strategy (see Results).

Heterologous expression of [FeFe]-hydrogenase alone is sufficient for small, measurable hydrogen production from *E. coli in vivo*, and this hydrogen production is increased with the co-expression of ferredoxins from several organisms [19]. Furthermore, overexpression of a ferredoxin oxidoreductase can link hydrogen production to cellular metabolism [20]. Pyruvate-ferredoxin oxidoreductase (PFOR) from several anaerobic or microaerobic species of microorganisms reduces ferredoxin as it breaks down pyruvate to acetyl-CoA. Coexpression of PFOR, ferredoxin, and [FeFe]-hydrogenase therefore couples the breakdown of glucose with the establishment of a reduced ferredoxin pool (Figure 1A). As [FeFe]-hydrogenases are efficient electron acceptors from ferredoxin, co-expressed [FeFe]-hydrogenases within this system can reset the redox state of the ferredoxin pool, thereby completing the circuit, and providing a readout of electron flux through the pathway as a function of hydrogen production.

Synthetic biology circuits such as the above-described PFOR-ferredoxin-hydrogenase pathway must be integrated into cellular metabolism in order to function, but must also be insulated from competing reactions in order to optimize output and ensure proper behavior. Natural electron transfer pathways are insulated in several ways that can be adapted for use in synthetic circuits (Figure 1B). Elimination of competing reactions, through gene deletion, transcriptional regulation, or spatial separation into different subcellular compartments, can isolate proteins from competition for electrons. For example, the expression of hydrogenase genes from many organisms are transcriptionally regulated by the presence of oxygen [21], likely in order to prevent competition for electrons with aerobic metabolism.

Isolation of electron transfer pathways can also evolve through physical changes to proteins, either through point mutations that alter interaction surfaces between two redox partners, or adaptations that co-localize interaction partners to one another or to secondary scaffolding complexes. The coevolution of protein interaction surfaces has been postulated to play a role in the control of bacterial signal transduction pathways [22] and is likely involved in the evolution of many iron-sulfur containing proteins capable of interacting with ferredoxins [23]. Isolation of iron-sulfur proteins through physical scaffolding, either in the mitochondrial or chloroplast membranes or through direct protein fusion, is thought to play a large role in the evolution of complex electron transfer pathways as well a the iron-sulfur proteins themselves. For example, larger [FeFe]-hydrogenases, such as those from *Clostridium *species contain several "ferredoxin-like" domains [24]. It is speculated that these domains arose through ancestral gene fusions, enhancing hydrogenase interaction with other ferredoxins, and providing an electron transport channel towards the hydrogenase active site.

Herein, we create and characterize a synthetic electron transfer circuit that couples hydrogen evolution with the breakdown of glucose in *E. coli *via heterologous expression of PFOR, Ferredoxin, and [FeFe]-hydrogenase. We used the modular nature of our synthetic circuit to recapitulate several of the aforementioned isolation strategies for electron transfer pathways. We reproduced spatiotemporal isolation through the deletion of competing iron-sulfur proteins. We explored the interaction surface of the hydrogenase and ferredoxin, testing four mutations of surface amino acids of the [FeFe]-hydrogenase from *Chlamydomonas reinhardtii *previously predicted to improve ferredoxin binding [25]. We synthetically modeled physical scaffolding of electron transfer proteins, both through direct protein fusion of the *Clostridium acetobutylicum *hydrogenase and ferredoxins with flexible peptide linkers, and through connection of hydrogenase and ferredoxin to a heterologous protein scaffold [26]. All of these insulation strategies significantly affected the function of our synthetic circuit, in many cases increasing total hydrogen production. The highest improvement was seen with direct protein-protein fusion of the hydrogenase and ferredoxin, with an optimal linker length increasing hydrogen production by up to four fold. This method is easily transferrable to other synthetic electron transfer pathways and may provide clues to understanding the evolution of complex electron transfer proteins with multiple ferredoxin-like domains.

## Materials and methods

### Plasmids and cloning

All cloning was done in *E. coli *DH5α. Hydrogenase genes from *Chlamydomonas reinhardtii *and the ferredoxin I gene from *Spinacia olearcea *were commercially synthesized by Codon Devices (Cambridge, MA), codon optimized for expression in *Saccharomyces cerevisiae *and acceptable for use in *E. coli *for wide applicability (see Additional file 1, figure S1 for nucleotide sequences). Hydrogenases from *Clostridium acetobutylicum *and *Clostridium saccharobutylicum *were cloned from plasmids received from Matthew Posewitz (National Renewable Energy Laboratory, Golden, CO). Hydrogenase genes HydA and HydB were cloned from *Shewanella oneidensis *using colony PCR of bacterial cultures from Colleen Hansel (Harvard University, Cambridge, MA). *Thermotoga maritima *HydA was cloned from genomic DNA provided by Kenneth Noll (University of Connecticut, Storrs, CT). PFOR and ferredoxin [27] were cloned from *Clostridium acetobutylicum *genomic DNA (ATCC, Manassas, VA). *Zea mays *ferredoxin was cloned from genomic DNA isolated from corn using DNeasy Plant Mini Kit (Qiagen, Valencia, CA). PFOR from *Desulfovibrio africanus *was isolated from plasmid pLP1 [28] provided by Laetitia Pieulle (Centre National de la Recherche Scientifique, Marseille, France) and *ydbK *was obtained through colony PCR of *E. coli *BL21. Plasmids for tetracycline-responsive expression of synthetic scaffold proteins were provided by John Dueber [26] (University of California, Berkeley), and ferredoxin/hydrogenase fusions to metazoan GBD, SH3, and PDZ ligand domains were constructed, bridged by flexible (Gly_4_Ser)_x _linkers. Oligonucleotides for metazoan ligand domains and linker sequences were purchased from Integrated DNA Technologies, with ligand sequences identical to those previously reported [26].

Site directed mutagenesis of the *Clostridium *and *Shewanella *hydrogenase genes to remove restriction sites needed for cloning and for alteration of *C. reinhardtii *HydA ferredoxin binding surface was carried out using the Quikchange Multi-Site Directed Mutagenesis Kit according to manufacturer instructions (Stratagene, La Jolla, CA). Cloning and mutagenesis primers are listed in Additional file 1, Table S1 (Integrated DNA Technologies, Coralville, IA)

Cloning of hydrogenase-ferredoxin fusion proteins was done using BioBrick standard assembly [29] and subsequently cloned into Novagen Duet vectors (Novagen, Gibbstown, NJ) whose multiple cloning sites were mutated to accept BioBrick parts (Additional file 1, figure S2). Strep-II tag (WSHPQFEK), (Gly_4_Ser) _2_, and (Gly_4_Ser) _4 _oligonucleotides with flanking BioBrick sites were purchased from Integrated DNA Technologies. Longer (Gly_4_Ser) linkers were made through BioBrick fusion of multiple linker sequences or through PCR amplification from other chimeric proteins [30].

### Protein expression

All protein expression and hydrogenase activity assays were performed in *E. coli *BL21 (DE3). Cells were transformed with modified pCDF-duet with *C. reinhardtii *HydEF in MCS1 and *C. reinhardtii *HydG in MCS2, and with modified pACYC-duet with *C. acetobutylicum *PFOR or *E. coli ydbK *in MCS1 or *Desulfovibrio vafricanus *PFOR cloned into the downstream NdeI and AvrII sites of MCS2. Hydrogenase/ferredoxin pairs were transformed either in each multiple cloning site of modified pET-duet, or for the *S. oneidensis *hydrogenase HydA in MCS1, HydB in MCS2, and ferredoxin in MCS1 of modified pCOLA-duet. To compare *in vitro *hydrogen production using maturation factors from *Clostridium acetobutilicum*, we used plasmids provided by Matthew Posewitz (pCDF-duet with CaHydE in MCS1, CaHydF in MCS2 and pET-duet with CaHydA in MCS1 and CaHydG in MCS2 [18]). Artificial scaffolds were expressed from pJD plasmids provided by John Dueber, previously described in Dueber et. al. 2009 [26].

### E. coli Gene Deletion

Hydrogenase knockout (Δ*hycE*, Δ*hyaB*, Δ*hybC*) and Δ*fpr*, Δ*ydbK*, Δ*hcr*, Δ*yeaX*, Δ*hcaD*, or Δ*frdB *single deletion strains were made by sequential P1 transduction from the Keio collection [31] into BL21(DE3) Δ*tonA*, followed by removal of the Kan^R ^marker by standard procedures.

### SDS-Page and Western Blotting

*E. coli *cells were lysed with Bacterial Protein Extraction Reagent (B-PER, Pierce, Rockford, IL), protein samples were normalized using the Bradford assay (Bio-Rad, Hercules, CA), diluted into SDS-PAGE loading buffer and loaded onto a 4-20% Tris/glycine/SDS acrylamide gel. α-Strep-tag II antibody (HRP-conjugated, Novagen, Gibbstown, NJ) or α-ferredoxin primary antibody (Agrisera, Vännäs, Sweden) and α-Rabbit IgG secondary antibody were used.

### Hydrogen production assays

Bacterial cultures were grown aerobically for two hours until reaching an OD_600 _of approximately 0.15 in LB media with appropriate antibiotic (50 μg/ml ampicillin, 25 μg/ml spectinomycin, 25 μg/ml kanamycin, and/or 12.5 μg/ml chloramphenicol) in 40 ml glass serum vials, induced with 1 mM IPTG (and 2 μg/ml anhydrous tetracycline when relevant for the induction of scaffold proteins) and sparged with argon. For the methyl viologen assay, adapted from King et. al. [18], vials were sparged for 2 hours and then lysed with assay buffer containing 50 mM Tris pH 7.0, 50% B-PER, 10 mM methyl viologen (Sigma, St. Louis, MO) and 50 mM sodium dithionite (Fisher, Pittsburgh, PA), the vials were capped with rubber septa, the cells were vortexed and allowed to rock overnight at room temperature. Hydrogen concentration in the headspace gas was measured by gas chromatography (Shimadzu GC-14A). *In vivo *hydrogen production assays were performed in a similar fashion, except that cultures were supplemented with 0.5% glucose at the time of IPTG induction, sparged for 30 minutes and simply capped and shaken ovenight at 37°C before measuring headspace gas composition. Glucose curves were measured in cells pretreated overnight with 1 mM IPTG then immediately diluted into LB + variable glucose + 1 mM IPTG, then sparged and grown overnight. All hydrogen production values were normalized to an OD_600 _of 0.15.

### Homology modeling

Homology model of *C. reinhardtii *HydA1 was made using the SWISS-MODEL [32] server with the *Clostridium pasteurinium *HydA X-ray structure (1FEH [24]) as a template.

## Results

### In vitro hydrogen production from heterologously expressed hydrogenases

To create a synthetic electron metabolism circuit with hydrogenase as the terminal electron acceptor, we first investigated the activity of various hydrogenase genes heterologously expressed in the presence of appropriate maturation factors. We adapted a previously established *in vitro *hydrogenase activity assay [18], and measured hydrogen production from crude lysates of bacteria expressing hydrogenases and maturation factors from several species in the presence of a chemical electron donor, methyl viologen. Previous reports have shown that the hydrogenase maturation factors from *C. reinhardtii*, HydEF and HydG, are unstable when heterologously expressed in *E. coli *[18], likely due to the genes' high GC content, while the maturation factors from *Clostridium acetobutylicum *were able to mature [FeFe]-hydrogenases from a wide range of species. Using commercially synthesized, codon optimized maturation factors from *C. reinhardtii *we were able to alleviate the instability of the gene constructs. We found that *in vitro *hydrogen production from the *Clostridium acetobutylicum *hydrogenase was identical when coexpressed with the synthetic maturation factors or with HydE, HydF, and HydG from *C. acetobutylicum *(data not shown). All subsequent experiments were performed using the optimized *C. reinhardtii *maturation factors.

**Figure 2:**
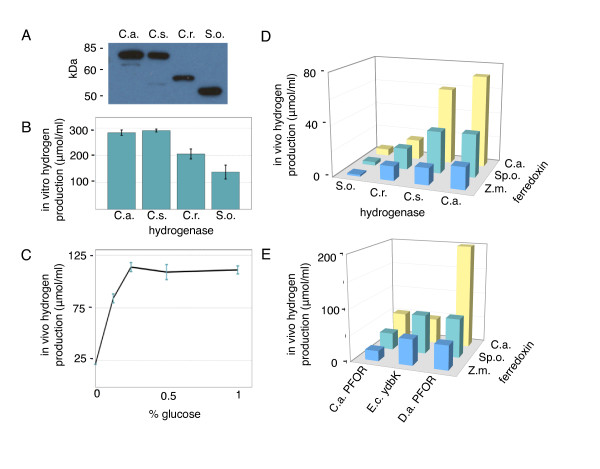
**Characterization of synthetic hydrogen production pathway **A.) Western blot of Strep-II tagged hydrogenase expression. B.) *In vitro *hydrogen production from *E. coli *strains expressing various hydrogenases, measured by the methyl viologen *in vitro *assay [18]. C.a. = *C. acetobutylicum*, C.s. = *C. saccharobutylicum*, C.r. = *C. reinhardtii*, S.o. = *Shewanella oneidensis. *C.) Glucose-dependence of hydrogen production. Here and below, *in vivo *and *in vitro *hydrogen production values are in units of μmol hydrogen/ml of *E. coli *culture, normalized to an OD_600 _of 0.15 unless otherwise stated. Assays were performed in triplicate, with error bars indicating standard deviation. D.) *In vivo *hydrogen production from *E. coli *strains expressing all combinations of the four hydrogenases vs. three ferredoxins from *C. acetobutylicum*, *Spinacia olearcea *(Sp.o), and *Zea mays *(Zm). E.) *In vivo *hydrogen production from the *C. acetobutylicum *hydrogenase paired with combinations of three ferredoxins and three PFOR genes.

We compared the *in vitro *hydrogen production of [Fe-Fe] hydrogenases from *Clostridium acetobutylicum, Clostridium saccharobutylicum, Chlamydomonas reinhardtii, Shewanella oneidensis*, and *Thermotoga maritima*, all of which are homologous in their catalytic domain (Additional file 1, figure S3). All hydrogenases except HydA from *Thermotoga maritima *could be expressed at a high level in *E. coli *(figure 2A), and were functional *in vitro *(figure 2B). Hydrogen levels increased linearly for the first several hours of measurement (data not shown), and we found that levels of hydrogen gas in the headspace after overnight incubation correlated to the relative rate of hydrogenase activity during this linear phase. Our overnight *in vitro *results agree with previous reports of *in vitro *hydrogen production rates, with the hydrogenases from *Clostridium *species producing the highest levels of hydrogen [18]. The heterologously expressed hydrogenase from *Shewanella oneidensis *is functional at relatively low levels *in vitro *when both subunits are coexpressed in *E. coli *with maturation factors from *C. reinhardtii.*

The *in vitro *assay is useful to test and compare the activities of heterologously expressed hydrogenase genes, but as the assay uses an exogenous reducing agent, it does not provide information on the electron flux within normal metabolic pathways *in vivo*. To measure electron flux *in vivo *as a function of hydrogen production, hydrogenase activity must be integrated into a functional electron transfer pathway. One well established class of electron donors to hydrogenases are ferredoxins, small soluble proteins that contain iron-sulfur clusters. Construction of a system where hydrogenase activity depends on electron transfer from ferredoxin would allow for comparison to *in vitro *data to provide information on hydrogenase behavior and hydrogenase-ferredoxin interaction dynamics.

### In vivo construction and optimization of a synthetic hydrogen-producing circuit

To produce hydrogen *in vivo *from glucose, the [FeFe]-hydrogenase was coexpressed with its required maturation factors, ferredoxin, and pyruvate-ferredoxin oxidoreductase (PFOR) from different species. In this heterologous circuit, PFOR oxidizes pyruvate to acetyl-CoA, reducing ferredoxin, which then transfers the electron to the hydrogenase. In normal *E. coli *metabolism, the oxidative breakdown of pyruvate to acetyl-CoA is performed either aerobically by the pyruvate dehydrogenase complex, reducing NAD^+^, or anaerobically by pyruvate formate lyase, generating formate (figure 1A). PFOR functions in certain anaerobic bacteria and in eukaryotic parasites that possess hydrogenosomes, organelles evolutionarily related to the mitochondrion that generate a proton gradient through the production of hydrogen gas [33]. PFOR is an attractive electron source for a synthetic hydrogen production circuit as overexpression of a putative *E. coli *PFOR homolog, YdbK, increases *in vivo *hydrogen production by heterologously expressed [FeFe]-hydrogenase and ferredoxin [20], PFOR purified from *Clostridium pasteurianum *has been shown to reduce a number of ferredoxins *in vitro *[34], and functional PFOR from *Desulfovibrio africanus *has been recombinantly expressed in *E. coli *[28].

**Figure 3:**
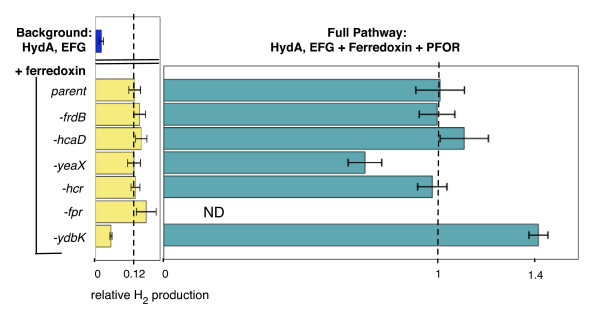
**Insulation of hydrogenase pathway through deletion of competing reactions **Relative hydrogen production of different knockout strains compared to parent strain (Δ*hycE*, Δ*hyaB*, Δ*hybC*) expressing hydrogenase alone (dark blue bar), hydrogenase and ferredoxin only (yellow bars) or the full PFOR-ferredoxin-hydrogenase pathway (green bars).

Consistent with the establishment of a synthetic electron transport circuit *in vivo*, we observed high levels of glucose-dependent hydrogen production upon coexpression of PFOR, hydrogenase and its maturation factors, and ferredoxin all from *Clostridium acetobutylicum *in an *E. coli *strain lacking endogenous hydrogenases (Δ*hycE*, Δ*hyaB*, Δ*hybC*, figure 2C). Hydrogen production was again measured after overnight incubation, as we found that hydrogen production *in vivo *from glucose was exhausted after 16 hours (data not shown). We were unable to detect hydrogen production in the parental strain of *E. coli *with the native hydrogenases deleted. Removal of any individual pathway component from the synthetic circuit drastically reduced *in vivo *hydrogen production. However, as has been previously reported, there was a small background level of hydrogen production from expression of hydrogenase and maturation factors alone [35]. Consistent with previous results [19], we found this background hydrogen production was slightly increased upon overexpression of ferredoxin in addition to hydrogenase, indicating that there are *E. coli *proteins capable of reducing both hydrogenases and plant-type ferredoxins, several candidate proteins of which we deleted in the following section (figure 3).

The hydrogenase-ferredoxin-PFOR pathway constitutes a modular system, where each element can be exchanged with homologous genes from different organisms. By coexpressing pathway enzymes from diverse microorganisms, we were able to compare the relative interaction strengths of four hydrogenases, three ferredoxins (figure 2D), and three PFORs (figure 2E). All ferredoxins were able to transfer electrons between PFOR and hydrogenases from different species with varying levels of efficiency.

*In vivo *hydrogen production from circuits expressing each of the four hydrogenases (*C. acetobutylicum, C. saccharobutylicum, C. reinhardtii*, and *S. oneidensis*) followed the same trend as the *in vitro *experiments, with the highest hydrogen production observed with the clostridial hydrogenases (figure 2D). The relative interaction and electron transfer rates for hydrogenase and ferredoxin were explored by comparing the *in vivo *hydrogen production of circuits made up from all pairwise combinations of the four hydrogenases and ferredoxin from *C. acetobutylicum, Spinacea olearcea*, and *Zea mays *and the PFOR from *C. acetobutylicum *(figure 2D). All hydrogenases produced the highest output when co-expressed with bacterial type 2-[4Fe-4S] ferredoxin from *Clostridium acetobutylicum*, with a potential of -420mV [27]. Intermediate levels of hydrogen were produced using leaf-type [2Fe-2S]-ferredoxin I from spinach, *S. olearcea *(-420 mV [36]) while the homologous root-type ferredoxin III from corn, *Z. mays *(-345 mV [36]) led to significantly lower *in vivo *hydrogen levels in all cases. Interestingly, the difference in hydrogen production from circuits expressing bacterial versus plant-type ferredoxins was more significant for hydrogenases from bacterial species. Hydrogenase from *C. reinhardtii*, which naturally pairs with plant-type ferredoxins, produced similar levels of hydrogen when co-expressed with ferredoxin from *C. acetobutylicum *or *S. olearcea *(figure 2D).

The interaction of overexpressed PFOR from *C. acetobutylicum, D. africanus*, or the PFOR homolog YdbK from *E. coli *with the three ferredoxins was compared in a similar fashion in circuits containing the *C. acetobutylicum *hydrogenase (figure 2E). Overexpression of PFOR from *C. acetobutylicum *and YdbK from *E. coli *led to similar levels of hydrogen production, although surprisingly, the highest levels of hydrogen produced from YdbK occurred when it was coexpressed with plant-type ferredoxin from *S. olearcea. *Overall, the highest levels of hydrogen production were seen with the PFOR from *D. africanus*, coexpressed with the hydrogenase and ferredoxin from *C. acetobutylicum.*

### Isolation of the hydrogen producing circuit through deletion of competing reactions

Natural biological electron transfer circuits are insulated to prevent electron leaks that can cause damage by creating oxygen radicals and insulated from one another to prevent "short circuiting" [37]. We sought to insulate our hydrogen producing circuit from competing metabolism to improve levels of hydrogen production and to better understand natural biological pathway isolation, a priority for the design of synthetic metabolic pathways. Although our constructed pathway is made up of genes that are divergent from *E. coli *metabolic enzymes, given the non-specific electrostatic interactions that mediate many ferredoxin interactions [5], native iron-sulfur proteins may interact with the proteins of the heterologous pathway. This is evidenced by the background hydrogen production in strains expressing only heterologous hydrogenases and ferredoxins (figure 3). Deletion of these potentially competing redox interaction partners should improve pathway function. To address these issues, we deleted six genes identified through their homology to plant-type ferredoxins or ferredoxin oxidoreductases that still allowed for viability (*fpr*, flavodoxin:NADP^+ ^reductase [19]; *ydbK*, the putative PFOR homolog [19]; *hcr*, an NADH oxidoreductase; *yeaX*, a predicted oxidoreductase; *hcaD*, ferredoxin:NAD^+ ^reductase; and *frdB*, fumarate reductase. Additional file 1, figure S4).

These six deletions were tested individually in a Δ*hycE*, Δ*hyaB*, Δ*hybC *background while expressing hydrogenase from *C. acetobutylicum *and maturation factors from *C. reinhardtii*, ferredoxin from *S. olearcea*, with or without co-expression of PFOR from *D. africanus*. Deletion of *fpr *and *ydbK *have been previously shown to slightly decrease the background level of hydrogenase activity *in vivo *[19]. We found that only the *ydbK *deletion had any significant effect on hydrogen production compared to the hydrogenase knockouts alone. The background level of hydrogen production from HydA and ferredoxin expressed alone was decreased by half in the *ydbK *deletion strain, whereas hydrogen production from the full pathway with the *D. africanus *PFOR was increased by 1.4 fold (figure 3). This is consistent with our finding that overexpression of *ydbK *led to high levels of electron transfer when co-expressed with ferredoxin from spinach, indicating that endogenous *ydbK *is able to disrupt the synthetic electron transfer pathway.

### Insulation through mutation of the hydrogenase-ferredoxin interaction surface

As an independent strategy, we attempted to insulate the pathway from competing electron metabolism through modification of the interaction surface of the hydrogenase and ferredoxin by rational protein design. Co-evolution of interacting protein pairs for preferential binding between natural partners likely plays a large role in the isolation of natural pathways [38], and this principle has been used in designing synthetic signal transduction systems [22]. To test whether mutations in a component of the artificial pathway specifically enhanced activity by improving the ferredoxin-hydrogenase interaction, we compared *in vivo *activity, which is ferredoxin-dependent, with *in vitro *activity, in which a chemical reducing agent, methyl viologen, drives hydrogen production. This *in vitro *assay thus measures ferredoxin-independent hydrogen production, reflecting the activity and expression level of the hydrogenase itself.

**Figure 4:**
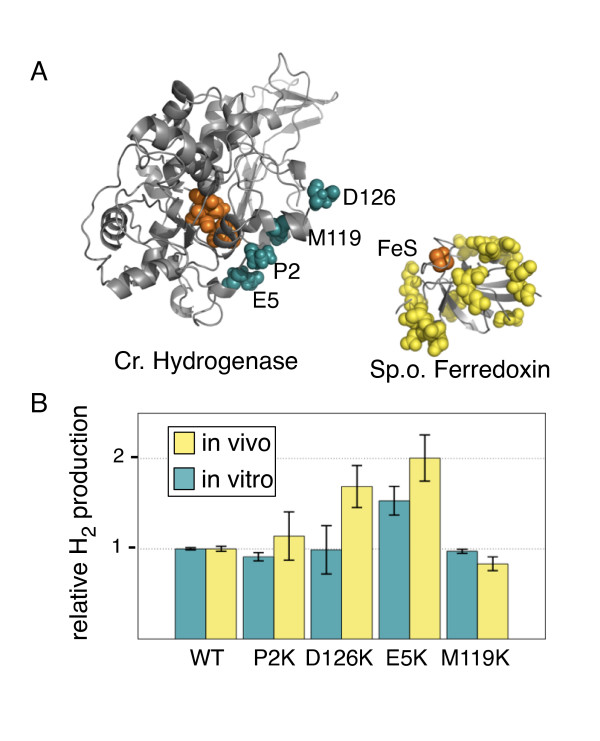
**Insulation of the hydrogenase pathway through ferredoxin binding surface mutagenesis **A.) Homology model of *C. reinhardtii *hydrogenase with mutated residues highlighted in cyan and spinach ferredoxin X-ray structure (1A70 [54]) with negatively charged residues highlighted in yellow. Iron-sulfur clusters and the hydrogenase catalytic cluster are highlighted in orange. B.) Relative *in vitro *and *in vivo *hydrogen production for wild type and mutated *C. reinhardtii *hydrogenase. Mutants D126K and E5K, which enhance charge-complementarity at the putative interaction surface, show a specific enhancement of *in vivo *activity relative to activity changes seen in the ferredoxin-independent *in vitro *assay.

The interaction between ferredoxins and clostridial hydrogenases is poorly characterized, with evidence that more than simple electrostatic reactions may play an role in mediating the transfer of electrons [34]. In contrast, the interaction surface between the hydrogenase from *Chlamydomonas reinhardtii *and its cognate ferredoxin has been extensively modeled *in silico*, with evidence that this interaction has a strong electrostatic component [39,40]. Long et. al. [25] proposed a structural model for the interaction between the hydrogenase HydA2 from *Chlamydomonas reinhardtii *and its cognate [2Fe-2S] ferredoxin, suggesting several mutations that might enhance this interaction due to improved charge complementarity (figure 4A). Ferredoxin is rich in negatively charged residues, and the mutations, E5K, P2K, M119K, or D126K are designed to increase the positive charge of the hydrogenase binding surface. We tested these mutations using HydA1 from *C. reinhardtii*, and ferredoxin from spinach, both of which are closely related to the proteins studied in the *in silico *model (Additional file 1, figure S5). We found that two mutations in HydA1, D126K and E5K, improved *in vivo *hydrogen production while *in vitro *these mutations showed less or no effect (figure 4B). As *in vitro *hydrogen production was closely correlated to hydrogenase expression level (data not shown), the improvement in *in vitro *hydrogen production that was seen for E5K may be the result of increased protein expression.

### Improvement of hydrogen production activity through direct protein fusion

**Figure 5:**
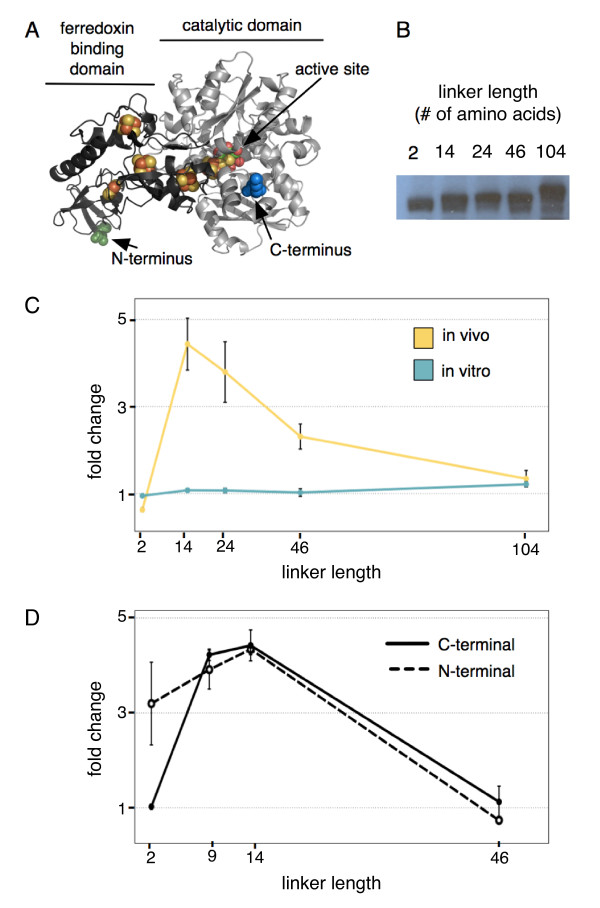
**Increase in hydrogen production by hydrogenase-ferredoxin fusion **A.) Schematic model of the protein fusion, here showing the *C. acetobutylicum *hydrogenase fused to the spinach ferredoxin N-terminus (N-termini highlighted in blue, C-termini in green, and iron-sulfur clusters in orange). B.) Hydrogenase-ferredoxin fusion proteins are highly expressed and are the predicted size for the chimera, as indicated by western blotting with an anti-ferredoxin antibody. C.) Linker-length dependent behavior of fusion with spinach ferredoxin to hydrogenase C-terminus *in vivo *or *in vitro*. D.) Linker-length dependent behavior of fusion with *Clostridium *ferredoxin at the hydrogenase N- or C-terminus *in vivo*.

Many metabolic electron transfer pathways are insulated through the physical scaffolding of protein components in the membrane, for example in the electron transport chain of mitochondria or chloroplasts. Additionally, some electron transport proteins themselves are built from combinations of modular electron binding domains, including the hydrogenase from *Clostridium *[24]. We sought to isolate our synthetic pathway and improve hydrogen production through physically linking the hydrogenase and ferredoxin in order to increase the chance of binding and electron transfer between the desired partners. We were able to show improved function of the artificial pathway through genetic fusion of the hydrogenase and ferredoxin. Chimeras between ferredoxin and various ferredoxin reductases have been shown to be functional *in vitro *[41], with improved electron transfer rates presumably due to the increased local concentration of reduced ferredoxin. We fused the hydrogenase from *C. acetobutylicum *(figure 5A) with ferredoxin from spinach (figure 5B & C) or from *C. acetobutylicum *(figure 5D) using flexible protein linkers of various lengths.

Our hydrogenase-spinach ferredoxin chimeric proteins were all expressed at a high level in *E. coli *(figure 5B) and active at identical levels *in vitro *(figure 5C), regardless of the orientation of the fusion or linker length, consistent with the lack of a requirement for ferredoxin in the *in vitro *assay. *In vitro *data for fusions with the *C. acetobutylicum *ferredoxin followed a similar trend (data not shown). However, *in vivo *activity of the fusion proteins when coexpressed with the PFOR from *D. africanus *depended on linker length as well as overall configuration. Fusions of the spinach ferredoxin to the hydrogenase C-terminus displayed decreased function when the linker is very short, a nearly five-fold improvement at intermediate length linkers, and activity falling gradually to the level of that of separate proteins at longer lengths. This length dependent behavior is consistent with models of changes in local concentration of reactants due to enzyme fusion [42]. Protein fusion to the C-terminus of the spinach ferredoxin, whether the hydrogenase or a short protein tag abrogated *in vivo *hydrogenase activity entirely (data not shown), likely due to the proximity of the ferredoxin C-terminus to the iron-sulfur cluster [41].

The behavior of the fusion protein is similar with the ferredoxin from *C. acetobutylicum*, although the bacterial ferredoxin is equally active when fused at either terminus (figure 5C). When fused to the hydrogenase C-terminus, the linker-length dependent activity displays similar characteristics to the spinach ferredoxin. Interestingly, when fused to the hydrogenase N-terminus, *in vivo *activity with shorter linker lengths is improved. The putative ferredoxin-binding region is on the N-terminal domain of the hydrogenase (figure 5A), which includes all of the F-clusters that transfer electrons from the surface to the active-site H-cluster. Fusion with a short linker at the C-terminus makes it impossible to reach the N-terminal domain, resulting in the decreased activity compared to unfused proteins. At the N-terminus, however, a short linker still allows for interaction between the hydrogenase and its fused ferredoxin, resulting in increased activity.

### The effect of scaffolding ferredoxin and hydrogenase

In signal transduction, complex protein scaffolds isolate pathways by localizing pathway components into a complex, directing the flow of information. These scaffolds can be rewired in their natural contexts in eukaryotic cells in order to alter the output behavior of signaling cascades [43,44]. Synthetic, modular scaffold proteins have been implemented in *E. coli *in order to direct flux in synthetic metabolic pathways, improving pathway output by up to 77-fold [26]. These synthetic scaffolds are built from modular scaffold domains from eukaryotic signal transduction--PDZ, SH3, and GBD domains--which tightly bind cognate ligand peptides that can be fused to any protein of interest for localization to the scaffold. We imported these scaffold designs into our synthetic pathway and found that scaffolding of the hydrogenase and ferredoxin dramatically affected the function of the pathway.

Scaffolding of metabolic pathways with small-molecule intermediates can lead to a "pipeline" effect, where the increase in the local concentration of upstream intermediates can speed up the reaction. This can significantly affect the pathway output, particularly when the chemical intermediates of the reaction pathway are toxic to the cell and increased throughput can lead to cell death if the intermediate is not rapidly converted by the next enzymatic step in the pathway [26]. Instead of small molecule intermediates, our synthetic pathway relies on protein-protein interactions, as is the case in many signal transduction cascades. By channeling electron transfer through scaffolded interactions, the flux through the synthetic circuit can potentially be significantly increased in an insulated manner. Because the tertiary structures of the synthetic scaffolds have not been determined, however, it is also possible that the requirement of protein-protein interaction for pathway function may lead to a decrease in pathway flux due to non-optimal insulation of interacting partners. We sought to characterize the effects of different scaffold structures, ligands, and linker lengths on the function of the synthetic PFOR-ferredoxin-hydrogenase circuit with ferredoxin and hydrogenase localized to the synthetic scaffold. All experiments were performed using the PFOR from *D. africanus*, and ferredoxin and hydrogenase from *C. acetobutylicum.*

The scaffold is an artificial protein built up of several modular peptide binding domains. The GBD binding domain is at the N-terminus, followed by the SH3 domain and the PDZ domain at the C-terminus. The number of domains in each case is variable, and in Dueber et. al., the ratio of the domains to one another made significant differences in flux depending on the stoichiometry of the reactions in the synthetic pathway [26]. Although multiple diffusion-limited metabolic pathways could be enhanced using this design [26], artificially scaffolded redox pathways have not yet been investigated. While we were primarily interested in exploring how different configurations of scaffold domains and linker lengths affected the interaction of the redox proteins and therefore hydrogen output, we measured a three-fold improvement of hydrogen production in the scaffolded vs. non-scaffolded conditions when all of the proteins were expressed off of the lower activity pTet promoter, leading to a decrease in the absolute value of hydrogen production when compared to the Duet vectors (data not shown).

**Figure 6:**
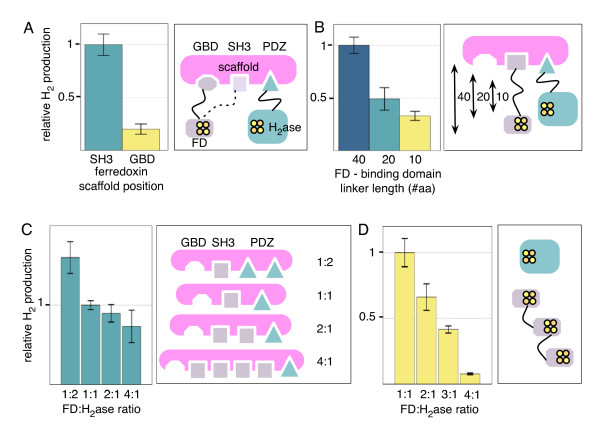
**Effect of artificial scaffolding configuration on hydrogen production from the synthetic circuit**. A.) Positional effects of ferredoxin targeting to artificial scaffold on hydrogen production. B.) Circuit efficiency is dependent upon length of flexible linker connecting ferredoxin (FD) to scaffolding. C.) Modulation of ferredoxin to hydrogenase ratio on scaffold affects hydrogen production, with decreasing yield observed at higher ferredoxin:hydrogenase ratios. D.) Direct fusion of ferredoxins to one another yields diminishing hydrogen production with increased numbers of fused ferredoxin proteins.

The ratio of the different scaffold domains, the ligand bound to the pathway components, and the length of the linker between the ligand and the ferredoxin protein all had significant effects on the output of the synthetic circuit (figure 6). Because ferredoxin and hydrogenase need to physically interact for the circuit to function, suboptimal configurations for the protein scaffold could conceivably sequester these proteins from one another. Indeed, we found that hydrogen output was decreased when the hydrogenase and ferredoxin were bound farther from one another along the length of scaffold (figure 6A). Furthermore, the length of the linker connecting ferredoxin with the SH3 ligand also significantly affected the ability of the hydrogenase and ferredoxin to interact while bound to the scaffold. Increasing the linker length from five amino acids to twenty led to a 3-5 fold increase in hydrogen output from the scaffolded circuit (figure 6B). Linkers of intermediate length produced intermediate pathway output.

The ratio of GBD, SH3, and PDZ domains that made up the synthetic scaffold also significantly affect the function of the pathway in some cases. The stoichiometry of the hydrogen production reaction requires two ferredoxins for the reduction of a single hydrogen molecule, so it might be expected that scaffolds that localize more ferredoxin molecules will increase flux through the hydrogenase. Unfortunately, however, PFOR is substrate limited [45], with increasing concentrations of ferredoxin leading to decreased enzymatic activity. Increasing the ratio of ferredoxin:hydrogenase bound to the scaffold decreases the output from the synthetic pathway (figure 6C). This effect was also seen when ferredoxin genes were fused in tandem without a scaffold, with output from the hydrogen production circuit steadily dropping with each added ferredoxin (figure 6D). This strategy may, however, increase pathway flux in other synthetic electron transfer pathways when the electron donor is not substrate limited.

## Discussion

Metabolic engineering to produce biofuels must necessarily involve the redirection of reducing equivalents into the fuel molecule and away from other cellular metabolites. In cells, reducing equivalents are primarily stored in iron-sulfur cluster proteins and in small molecules such as NADPH, NADH, and FADH_2_. While the small molecules can freely diffuse through the cell and interact with a wide variety of enzymes, iron-sulfur proteins can be isolated through the techniques of metabolic and protein engineering. In the experiments described here, we applied four approaches to controlling electron flow out of the iron-sulfur cluster protein ferredoxin: deletion of potential interaction partners, enhancement of interaction by engineering of a protein surface, and increasing the local concentration of interacting proteins using a flexible peptide linker or attachment to a scaffold protein.

To measure the effect of these approaches, we first created an artificial pathway to produce the biofuel hydrogen in *E. coli*. The pathway consists of the proteins pyruvate-ferredoxin oxidoreductase (PFOR), ferredoxin, and a hydrogenase (expressed in the presence of hydrogenase maturation factors). This pathway produces a theoretical maximum of two molecules of hydrogen per input glucose, and still allows acetyl-CoA production from pyruvate. We characterized the relative efficacy of hydrogen production using various combinations of PFOR, ferredoxin, and hydrogenase molecules from different species, and found that PFOR from *D. africanus *in combination with ferredoxin and hydrogenase from *C. acetobutylicum *was the most active pathway, predicted in part by previous *in vitro *data [18,34,46].

To direct electron flow from ferredoxin into hydrogenase, we first deleted genes encoding six other proteins with which PFOR and/or ferredoxin might interact. Of these, only deletion of *ydbK*, encoding a putative *E. coli *PFOR, resulted in enhanced hydrogen production. In addition, in the absence of the PFOR from *D. africanus*, deletion of *ydbK *resulted in a decrease in the background level of hydrogen. These results provide further evidence that *ydbK *is a functional PFOR that can interact with a variety of electron acceptors, particularly the spinach ferredoxin [20].

As a converse approach, we addressed whether the levels of hydrogen production could be enhanced by improving the binding between ferredoxin and hydrogenase *in vivo*. In this case we used the *C. reinhardtii *hydrogenase, whose interaction with plant-type ferredoxin has been computationally modelled by Long et al., who suggested mutations that could enhance this interaction [25]. Several mutations that improve charge complementarity between spinach ferredoxin and this hydrogenase were found to enhance electron transfer between these proteins *in vivo*, as inferred by increased hydrogen production. These results indicate that the activity of the hydrogenase is limited, in part, by its ability to interact with ferredoxin; i.e. that the collision and docking of these proteins is not effectively in excess.

Finally, we used two different methods to increase the relative local concentration of ferredoxin and hydrogenase: direct fusion by a flexible glycine/serine-rich linker, and indirect fusion by attachment of these proteins to interaction modules that bind to a common scaffold. Each approach significantly improved hydrogen production in a strongly configuration-dependent manner *in vivo*, as expected if the ferredoxin and hydrogenase were primarily interacting in cis. For example, when the ferredoxin and hydrogenase were attached by a linker too short to allow in cis interaction, hydrogen production was relatively low, but increased significantly when the linker was long enough to allow interaction. As the attaching linker was further lengthened, hydrogen production decreased gradually, consistent with the two proteins occupying a sphere of increasing volume and decreasing relative concentration. As a tactic for metabolic engineering, protein fusion and/or scaffolding is particularly useful with iron-sulfur cluster proteins, because their electrons must be transferred protein-to-protein--no small molecule carriers of reducing equivalents are generated that might diffuse away.

The iron-sulfur proteins in our synthetic circuit present a modular system, with proteins from disparate species able to interact and produce high levels of hydrogen. Such modular systems are valuable for further synthetic biological manipulation and experimentation. The synthetic pathway presented here is a relatively simple method for the analysis of activities and electron transfer properties of hydrogenases, ferredoxins, and PFOR genes from any number of species, or engineered synthetic electron transfer proteins. These *in vivo *data are a valuable complement to *in vitro *binding constants and kinetic parameters of the enzymes and will be useful in further designing and optimizing microbial systems for hydrogen production.

Such synthetic biological systems can also be used to better understand biological electron transfer systems. The role of ferredoxins in *E. coli *metabolism is poorly characterized, with ferredoxins performing many unknown but required functions in the cell. Here we tested deletions of six iron-sulfur proteins expected to interact with ferredoxins, many of which are previously uncharacterized. While only one gene deletion (Δ*ydbK*) affected our specific hydrogen production pathway, combinatorial deletions may affect hydrogen production in different ways, or may affect other synthetic electron transfer pathways. Further deletions of iron-sulfur oxidoreductases and combinations thereof may lead to a more complete understanding of electron transfer systems in the *E. coli *cytoplasm, as well as the development of a host strain for expression of heterologous electron transfer pathways for synthetic biology. Such a strain would have to retain the ability to mature iron-sulfur clusters but limit the function of proteins that can interact with ferredoxins and ferredoxin oxidoreductases to ensure optimal electron flux through the synthetic pathway. Such specialized strains of *E. coli *may be optimized for other types of synthetic pathway designs and may be better equipped for industrial purposes than proposed "minimal" cells [47], as they would retain many of the mechanisms that allow for robust growth and protein expression.

Our pathway can also be used to further analyze protein-protein interaction surfaces for electron transfer, including for mutagenic studies to determine the binding surface on the clostridial hydrogenases, which is poorly understood. An improved understanding of the electron transfer surface between the hydrogenase and ferredoxin would significantly affect our picture of how electron transfer pathways co-evolved; whether specific ferredoxins evolved for interaction with specific enzymes or whether electron transfer is regulated in other ways. Considering the ability of ferredoxins from many distant species to interact with various hydrogenases, the impact of further binding surface optimization may be negligible, or may require co-evolution of complementary mutations on both binding partners to result in highly specific interactions.

Iron-sulfur proteins are also uniquely suited to isolation techniques that involve physical scaffolding. Electrons tunnel between proteins in close proximity, so direct protein fusion improves the local concentration of electron transfer proteins and thus improves the electron transfer rates. This is in contrast to other synthetic metabolic pathways with small molecule intermediates, whose diffusion through the cellular environment is much faster, limiting the potential improvement by protein fusion. This method can be easily adapted to other electron transfer pathways in a modular, extensible manner. Moreover, the dependence of hydrogenase activity upon scaffold design and/or the links between the ligands and scaffold illustrates that synthetic redox pathways can be coupled through interaction with a common adaptor protein in order to modulate electron flux through the system. Unlike reactions with diffusible intermediates, scaffolding of redox partners requires that the scaffold design allow sterically unhindered interaction between bound protein to enable electrons to tunnel between closely apposed iron-sulfur clusters. Incorrect design may tether redox partners in a manner that constrains them and prevents electron transfer, as we observed when hydrogenase and ferredoxin were bound to domains on distal ends of the scaffold. Through attention to scaffold design, further optimization may significantly improve hydrogen production through the synthetic circuit, as well as provide a template for future scaffolding of other electron transfer pathways.

Biological hydrogen production is a promising and well-studied system for sustainable energy production. The insulation approaches presented here are widely applicable to other biological hydrogen systems, from *in vitro *enzymatic pathways [11], where protein fusions would likely improve kinetic rates, to methods for boosting natural hydrogen production in heterotrophic [48] and photosynthetic [49,50] organisms. Photosynthetic systems in particular may benefit from the insulation strategies presented here, as ferredoxins are the primary electron carrier in photosynthetic organisms and competition for electrons from other metabolic pathways is strong. Improvement of protein binding in plant-type ferredoxins may be useful in such systems, as well as the applicability of protein fusion or pathway scaffolding to a wide range of biological systems.

The three methods described here - deletion of competing reactions, optimization of interaction surfaces, and protein tethering - could be combined with each other and with other strategies for optimizing redox pathways. Direct fusion of proteins with a flexible linker had the largest effect by itself, and simultaneous deletion of competing reactions should have a multiplicative effect since these involve independent aspects of the pathway. Combining protein fusion with optimization of protein interaction surfaces may have a sub-multiplicative effect because both approaches affect the same pathway step.

Alone, the pathway presented here has a maximum theoretical yield of 16.67%, with two hydrogen molecules produced for every molecule of glucose. Our *in vivo *hydrogen production system produced hydrogen at levels that ranged from 0.005% to nearly 3% of this theoretical maximum. Combination of methods presented here with other metabolic engineering approaches will likely increase hydrogen production from pyruvate closer to the theoretical yield. In particular, a background strain with deletions in several genes that either take electrons away from the pathway, limit iron sulfur cluster biogenesis, or use glycolytic flux from pyruvate would drastically increase hydrogen production. Such an engineered strain would be have deletions in *ydbK*, shown here to boost hydrogen production, as well as in *iscR*, a negative regulator of iron sulfur cluster biogenesis shown to limit hydrogen production from heterologously expressed [FeFe]-hydrogenases [35]. Flux through PFOR could be increased through the deletions of pyruvate metabolism genes such as the pyruvate dehydrogenase complex and pyruvate formate lyase, as well as other metabolic enzymes that consume pyruvate, which when deleted have led to accumulation of large quantities of pyruvate in *E. coli *[51]. Addition of optimized hydrogen production through natural [NiFe]-hydrogenase pathways, deletion of uptake hydrogenase genes [52], and incorporation of other ferredoxin-oxidoreductases that generate electrons from other metabolic conversions [19] is likely to bring hydrogen production closer to the absolute theoretical maximum of 12 moles of hydrogen per mole of glucose.

## Conclusions

Electron transfer systems such as our hydrogenase pathway are an untapped resource for synthetic biology, which seeks to design biological pathways as predictably as electronic circuits [53]. Electrons are unique metabolites whose movement in biological systems occurs by quantum-mechanical tunneling between protein-bound cofactors such as iron-sulfur clusters. As a result, escape by diffusion into an aqueous phase is avoided, offering distinctive opportunities for control. The circuit described here moves electrons from higher to lower energy, while performing work in the form of hydrogen production. The rationally constructed insulation of the pathway through elimination of side reactions, interaction surface optimization, and protein fusion or scaffolding indicate that all four methods are viable for synthetic circuit design and all strategies may play a role in the evolution of complex isolated circuits in natural metabolism. This type of synthetic-biological analysis may yield insights into natural mechanisms for controlling electron flow, and may provide new approaches for metabolic engineering and bioenergy.

## Competing interests

The authors declare that they have no competing interests.

## Authors' contributions

CMA, DCD, PMB, EHW, JCW, and PAS designed experiments and analyzed data; CMA, DCD, PMB, EHW performed experiments; CMA, DCD, JCW, and PAS wrote the paper. All authors read and approved the final manuscript.

## Supplementary Material

Additional file 1 **Supplementary information for Agapakis et. al. "Insulation of a synthetic hydrogen metabolism circuit in bacteria." **Supplementary information includes nucleotide sequences for commercially synthesized genes, a table listing all oligonucleotide primers used, sequence information for the mutated expression vectors, protein sequence alignment of hydrogenase enzymes used in the hydrogen metabolism circuit, domain structure of electron transfer proteins deleted in the study, and nucleotide alignment of *Chlamydomonas reinhardtii *HydA1 and HydA2. Click here for file
