# Supplementary material for: Insulation of a synthetic hydrogen metabolism circuit in bacteria
Source: J Biol Eng. 2010 Feb 25;4:3. doi: 10.1186/1754-1611-4-3 (PMC2847965; doi:10.1186/1754-1611-4-3)
Supplement: Additional file 1 — Supplementary information for Agapakis et. al. "Insulation of a synthetic hydrogen metabolism circuit in bacteria." Supplementary information includes nucleotide sequences for commercially synthesized genes, a table listing all oligonucleotide primers used, sequence information for the mutated expression vectors, protein sequence alignment of hydrogenase enzymes used in the hydrogen metabolism circuit, domain structure of electron transfer proteins deleted in the study, and nucleotide alignment of Chlamydomonas reinhardtii HydA1 and HydA2. [file 1754-1611-4-3-S1.PDF]

Supplementary Information for Agapakis et. al. “Insulation of a synthetic hydrogen metabolism circuit in bacteria”

```
>Cr.HydA1;AAL23572
gaattcgcggccgcttctagagctgcaccagccgcagaagctcctttgtctcatgttcaacaggccttag
ccgagcttgcaaaaccaaaggatgaccctactagaaaacacgtatgtgtccaagtggccccagctgttag
ggtagcaattgctgaaacacttggtttggcccctggagcaaccactccaaagcagttagctgagggccta
agaaggcttggttttgatgaagtgttcgacacattgtttggagccgatttaaccataatggaagagggct
cagaattgttacatagactaactgaacaccttgaggcacatcctcactccgacgaaccattgcctatgtt
cacaagttgctgtccaggttggatcgctatgttagaaaaaagctatcctgatctaattccatacgtgagc
tcatgcaagtcccctcaaatgatgttggccgcaatggttaaaagttatttagctgagaagaaaggtatag
ccccaaaggatatggtaatggtcagcatcatgccatgtaccagaaaacaatctgaagcagacagggattg
gttttgcgttgacgctgatcctactcttagacagttggatcatgtgattacaaccgttgagttaggaaat
atattcaaggaaagaggcatcaacctagccgaacttccagagggtgaatgggacaatcctatgggagtag
gttcaggcgcaggtgtcttgtttggaactacaggcggcgtgatggaagctgctttaaggactgcctacga
gctattcaccggtacaccattgcctagattatcccttagtgaagttaggggaatggatggtattaaagaa
actaacattaccatggtaccagcacctggctctaagtttgaggaattgttaaaacatagagctgccgcaa
gagctgaagccgcagctcacggaacaccaggtcctctagcatgggacggcggtgctggattcactagcga
ggatggtaggggcggcataacattgagagtcgccgttgcaaatggattaggtaacgctaaaaagcttatc
accaaaatgcaagccggcgaagcaaagtatgattttgtggagattatggcttgtccagccggatgtgttg
gtggaggcggacaacctagatcaactgacaaagcaataacacagaagaggcaagctgccctatacaattt
ggatgaaaaatccactttaagaagaagtcatgaaaacccatctatcagggagctttatgacacctacttg
ggtgaacctttaggtcacaaggcacatgaactattgcacacacattatgtagctggcggagtcgaggaaa
aagatgaaaagaaaactagtagcggccgctgcag
```

```
>Cr.HydEF;AAS92601
gaattcgcggccgcttctagagctgcacatgcctctgcttcaaaagcaactccagatgttcctgtagacg
atcttccacctgcccacgctagagcagccgtcgccgcagctaataggagagccagggcaatggcttccgc
cgaagcagctgccgagacattaggtgactttctaggacttggcaagggtggattgagtccaggcgcaacc
gctaacttagatagagaacaagtgctaggtgttcttgaggccgtatggagaaggggtgacttgaatttag
aaagagcattgtatagccatgctaacgccgtcactaataaatactgtggaggcggtgtgtattacagagg
attagttgagttctctaacatttgccagaatgattgttcatattgcggtataaggaacaatcaaaaggag
gtatggagatacacaatgcctgtcgaagaagttgtggaggttgcaaaatgggccctagaaaacggcatca
ggaatattatgcttcagggtggagaacttaagaccgagcaaagattagcttacctagaagcctgtgtaag
agcaataagggaggaaactacacaattggatttagaaatgagagctagagccgcatccaccactacagct
gaggccgcagctagtgcacaggctgacgccgaagcaaaaaggggtgaaccagagcttggcgtcgtggtta
gcttgtctgtaggtgaattacctatggaacaatacgagagactatttagagctggagccaggagatatct
tatcaggattgaaacctcaaatccagatttgtacgcagctttacaccctgaaccaatgtcctggcatgcc
agagtcgagtgcctaagaaacttgaagaaagcaggttatatgttaggcactggagttatggtgggccttc
ctggccaaacattgcacgacttagctggtgatgttatgttctttagggatataaaggccgacatgatcgg
aatgggtccattcattactcagcctggcaccccagcaacagataaatggactgctctatacccaaatgct
aacaagaatagtcatatgaaatctatgtttgacttgaccacagccatgaacgcattagtaagaattacta
tgggtaatgtcaacataagcgctacaaccgcccttcaagcaatcattcctactggaagagaaatagccct
agagaggggtgccaatgtggttatgccaatcttgacacctactcagtatagagaatcataccaattatat
gaaggcaagccatgtattaccgatacagcagtacaatgtagaaggtgccttgatatgagattgcattccg
tcggaaaaaccagtgctgccggtgtttggggtgaccctgcatctttcttacacccaatagtgggcgttcc
tgtaccacatgatctatcatctcctgctttggccgcagctgccagcgcagactttcacgaggtcggagct
ggtccatggaaccctatcaggttagaaagacttgttgaagtgccagatagataccctgatccagacaatc
atggtaggaaaaaggccggcgcaggaaaaggcggcaaggctcacgattcccatgacgatggagatcatga
cgatcaccatcaccatcatggtgccgcaccagctggtgccgcagctggcaaaggaaccggtgccgcagct
attggcggcggagccggtgctagcagacagagagtagctggcgccgcagctgcctcagcaaggttgtgtg
ctggagccagaagagcaggtagggtcgttgcttctcctctaagaccagccgcagcttgcaggggtgtggc
cgttaaggcagctgctgccgcagctggcgaggacgccggagcaggtacaagcggtgtaggctccaatatt
gtcaccagtcctggaatagcttcaaccacagcccacggtgttccaagaatcaacattggcgtgttcggag
taatgaatgcaggtaaatctactttagtcaacgctttggcccaacaagaagcatgtatagttgatagcac
ccctggtacaactgctgacgtcaagaccgttcttttagaactacatgcattgggcccagctaaattactt
```

gatacagccggattggatgaggtaggtggtctaggcgacaagaaaagaaggaaggcattaaatactttga
aagaatgcgatgtcgctgttcttgtggtagacaccgatacagccgcagctgccatcaaatccggaagatt
agcagaggccctagaatgggaaagtaaggtcatggagcaggctcacaaatacaacgtttcacctgtgttg
ttattgaatgtaaagagcagaggccttccagaagcccaagcagccagcatgctagaagccgttgcaggca
tgttagatccttccaaacagattccaaggatgtcattggacttagcttctactcctcttcatgagagaag
tacaataactagcgcctttgtcaaggaaggagcagttaggtcctcaagatacggtgctccactacctggt
tgtttgccaagatggtctttaggcaggaacgccagattgcttatggtgattccaatggatgcagaaaccc
ctggaggtagactattaaggccacaagctcaagtaatggaggaagccatcagacactgggcaacagtctt
gagtgttagattagacttggatgctgccaggggtaaacttggccctgaagcatgtgagatggaaagacag
aggttcgatggagtaattgctatgatggagagaaatgacggtccaactctagttgtgaccgattctcaag
ccatagacgtcgttcatccttggacattagatagatcctcaggcaggccattggtgcctatcactacctt
tagtattgcaatggcttatcaacagaacggaggtagacttgatccatttgtagaaggcctagaagcctta
gagacattgcaagacggcgatagagtcttaatatctgaagcatgcaatcataataggatcacttcagctt
gtaacgacattggaatggttcaaatacctaataagttggaagctgcacttggtggtaaaaagctacagat
tgagcacgctttcggcagagaatttccagaattagagtctggaggtatggatggcttgaaacttgccatc
cattgcggaggttgtatgattgatgcacaaaagatgcagcaaagaatgaaagacctacacgaagctggtg
tacctgttaccaactatggcgtgttctttagctgggccgcatggccagatgctttaaggagagccttgga
accttggggagtcgagcctccagttggtacacctgcaactccagctgccgcacctgctaccgccgcatcc
ggtgtgactagtagcggccgctgcag

>Cr.HydG;AAS92602
gcggccgcttctagaactgctcatggtaaagcatctgccacaagagaatatgctggagatttttgccag
gcaccactatttcacacgcatggtccgttgagagggaaacacatcacagatacaggaatcctgccgagtg
gataaacgaagctgcaatccataaggccttagaaaccagtaaagctgacgcacaagatgctggtagagta
agagagattctagccaaggcaaaagaaaaggctttcgtcactgaacacgccccagtgaatgcagagagca
aatctgaatttgttcagggacttacattggaagagtgtgctaccttaataaacgtagactcaaataacgt
cgaactaatgaatgagatcttcgatactgcccttgcaattaaggaaaggatatatggcaacagagtggtt
ttgtttgctcctttatacatcgccaatcattgcatgaacacatgtacctattgcgcattcagatccgcta
ataaaggtatggaaaggagtattttgactgacgatgatttaagagaggaagtagccgcactacaaaggca
gggtcatagaaggattcttgctttgacaggagaacacccaaagtacacttttgacaatttcttacatgct
gtcaacgttatagccagcgtgaaaaccgagcctgaaggctctatcaggagaattaatgttgaaatcccac
ctctatcagtatccgatatgagaaggttgaagaacacagacagtgtcggtacttttgtgttattccaaga
gacctatcacagagatacatttaaagttatgcatccatctggacctaagagcgatttcgactttagagta
cttactcaagatagggcaatgagagctggtttggacgatgtcggcatcggtgccttatttggactatacg
attataggtacgaagtttgtgcaatgcttatgcactcagaacatttggagagagaatataatgctggtcc
acatacaatttccgtgcctagaatgaggccagccgacggcagtgagttatctatagcacctccatacca
gttaacgatgctgacttcatgaagctagtagcagtcttgagaatcgctgtgccttataccggtatgattt
tatcaactagagaatctccagaaatgaggagcgcccttttgaaatgcggaatgtcccagatgagtgcagg
ttcaagaacagatgttggcgcttaccacaaggatcatactttatctaccgaggccaatctaagcaaattg
gcaggacaatttacattacaagacgaaagacctactaacgaaattgtaaagtggcttatggaggaaggtt
atgtcccatcctggtgtaccgcttgttacaggcagggcagaacaggtgaagatttcatgaatatatgcaa
agccggagacatccacgattttgtcatcctaacagtctattgactttacaagagtatcttatggattac
gcagacccagatttgaggaagaaaggtgaacaggttattgctagagagatgggccctgacgcctcagaac
cattatctgcacaaagcagaaagaggctagaaagaaaaatgaagcaagtgttggagggtgaacatgatgt
ttatttaactagtagcggccg

>So.Fd;1704156A
gcggccgcttctagagctgcatataaagttactttggtaacaccaaccggtaatgtcgaatttcaatgtc
ctgatgacgtgtacattttagacgccgctgaggaagagggaatagatctaccatattcttgcagagcagg
ctcatgttccagttgcgccggtaagcttaaaactggaagcttgaaccaggatgaccaatctttcttagat
gatgaccagatcgatgaaggctgggttctaacatgtgctgcataccctgtatcagacgtcaccattgaaa
ctcataaggaggaagaacttacagccactagtagcggccg

Figure S1. Sequence of codon optimized commercially synthesized genes. Gene names listed as Organism.Gene Name; GenBank Accession Number (Cr=*Chlamydomonas reinhardtii*, So=*Spinacia olearcea*)

Gene	GenBank Accession	Primer (Italics indicate Biobrick sites)
Ca HydA 5'	CAC3230	*CCTTTCTAGA* ATGAAAACAATAATCTTAAA
Ca HydA 3'		*AAGGCTGCAGCGGCCGCTACTAGT* TTCATGTTTTGAAACATTTTTA
Cs HydA 5'	CAU09760	*CCTTGAATTCGCGGCCGCATCTAGA* ATGATAAACATAGTAATTGATGAAA
Cs HydA 3'		*AAGGCTGCAGCGGCCGCTACTAGT* TTTATTGTATTTTAAGTGTAATAAT
So HydA 5'	SO_3920	*CCTTGAATTCGCGGCCGCATCTAGA* ATGACAACGACAACTTATCAACCAG
So HydA 3'		*AAGGCTGCAGCGGCCGCTACTAGT* AATGTTACCCAGCCATGAAGA GCCT
So HydB 5'	SO_3921	*CCTTGAATTCGCGGCCGCATCTAGA* ATGAACAAGAAAAAACACCTATTTG
So HydB 3'		*AAGGCTGCAGCGGCCGCTACTAGT* AGAGCTTAATTTGGTGCGATCGACA
Tm HydA 5'	AF044577	*CCTTGAATTCGCGGCCGCATCTAGA* ATGAAAATTTACGTTGATGGAAGAGAAGTTAT
Tm HydA 3'		*AAGGCTGCAGCGGCCGCTACTAGT* TCAGCCATTTTTCGAAAGCTCCTCCAGCACCTTCTC
Ca PFOR 5'	CAC2229	*CCTTGAATTCGCGGCCGCATCTAGA* ATGAAAAAAATGAAAACTATGG
Ca PFOR 3'		*AAGGCTGCAGCGGCCGCTACTAGT* TTATTGATTAGCTAATCTTT
Da PFOR 5'	CAA70873	AAACATATGGGAAAGAAAATGATGACG
Da PFOR 3'		AAACCTAGGCTACTTCTTCGTCCGCTTGC
Ec ydbK 5'	CAQ31878	*CCTTGAATTCGCGGCCGCATCTAGA* ATTACTATTGACGGTAATGGCG
Ec ydbK 3'		*AAGGCTGCAGCGGCCGCTACTAGT* TTAATCGGTGTTGCTTTTTTCCGC
Ca Fd 5'	CAC0303	*CCTTGAATTCGCGGCCGCATCTAGA* ATGGCATATAAAATAACAGA
Ca Fd 3'		*AAGGCTGCAGCGGCCGCTACTAGT* CTCTTGAACTGGAGCTCCTA
Zm Fd 5'	EU956042	*CCTTGAATTCGCGGCCGCATCTAGA* ATGGCTGTATACAAGGTGAAGCTTG
Zm Fd 3'		*AAGGCTGCAGCGGCCGCTACTAGT* CAGGTCGCCTTCCTTGTGGGTGTGG

Ca mut. 5'	GTGGACAATGTTCCAGAAGAGAAAATTGTGAGTTCCTTAAACTTG
Cs mut. 5'	GCAGCAAACTTAGCTGAGTTCATGAATAGCGG
SoB mut.	CGCGGTAGAAAAGTACTACAAAGAGTTCGGTGGCGAGCCATTAGGACATATGTCCC
CrHydA P2K	CCGCTTCTAGAGCTGCAAAGGCCGCAGAAGCTCCTTTGTC
CrHydA D126K	CGCTATGTTAGAAAAAAGCTATCCTAAGCTAATTCCATACGTGAGC
CrHydA E5K	CCGCTTCTAGAGCTGCACCAGCCGCAAAGGCTCCTTTGTC
CrHydA M119K	CCAGGTTGGATCGCTAAGTTAGAAAAAAGCTATCC

Table S1: Primers used for cloning and mutagenesis of heterologous pathway components

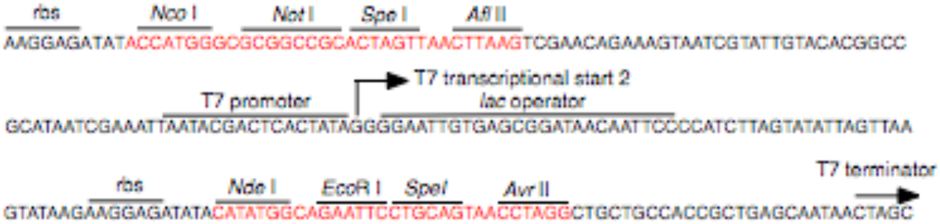

Figure S2. Sequence of modified multiple cloning sites of Novagen Duet Vectors. 5' phosphorylated oligonucleotide inserts (Integrated DNA Technologies, red text) were inserted between *Nco* I and *Afl* II sites in MCS1 and *Nde* I and *Avr* II of MCS2 of Novagen Duet Vectors pET-Duet, pACY-duet, pCDF-duet, and pCOLA-duet for heterologous expression of up to eight BioBrick sequences

```
Ca      ---MKTIIINGVQFNTDEDTTILKFARDNNIDISALCFLNNCNNDINKCEICTVEVEG-T 56
Cs      ---MINIVIDEKTIQVQENTTVIQAALANGIDIPSLCYLNECGN-VGKCGVCAVEIEGKN 56
Cr      ------------------------------------------------------------
So      MNKKKHLFAEDSFFLSRRKFMAVGAAFVAALAIPIGWFT--------------------S 40
Tm      ---MKIYVDGREVIINDNERNLLEALKNVGIEIPNLCYLSEASIYG---ACRMCLVEING 54

Ca      GLVTACDTLIEDGMIINTNSDAVNEKIKSRISQLLDIHEFKCGPCNRRENCEFLKLVIKY 116
Cs      NLALACITKVEEGMVVKTNSEKVQERVKMRVATLLDKHEFKCGPCPRRENCEFLKLVIKT 116
Cr      ------------------------------------------------------------
So      KLERRNEYIKARSQGLYKDDSLAKTRVSHANPAVEKYYKEFGGEPLGHMSHELLHTHFVD 100
Tm      QITTSCTLKPYEGMKVKTNTPEIYEMRRNILELILATHNRDCTTCDRNGSCKLQKYAEDF 114

Ca      KARASKPFLPKDKTEYVDERSKSLTVDRTKCLLCGRCVNACGKNTETYAMKFLNKNGKTI 176
Cs      KAKANKPFVVEDKSQYIDIRSKSIVIDRTKCVLCGRCEAACKTKTGTGAISICKSESGRI 176
Cr      ------------------------------------------------------------
So      RTKLSSMTTTTYQPGEIQG---LIKINASKCKGCDACKQFCPTHAINGASGAVHS----- 152
Tm      GIRKIR--FEALKKEHVRDESAPVVRDTSKCILCGDCVRVCEEIQGVGVIEFAKRGFESV 172

Ca      IGAEDEKCFDDTNCLLCGQCIIACPVAALSE-KSHMDRVKNALNAPEKHVIVAMAPSVRA 235
Cs      VQATGGKCFDDTNCLLCGQCVAACPVGALTE-KTHVDRVKEALEDPNKHVIVAMAPSIRT 235
Cr      ------------------APAAEAPLSHVQQALAELAKPKDDPTRKHVCVQ--VAPAVRV 40
So      --------IDEDKCLSCGQCLINCPFSAIEETHSALETVIKKLADKNTTVVGIIAPAVRV 204
Tm      VTTAFDTPLIETECVLCGQCVAYCPTGALSI-RNDIDKLIEALES-DKIVIGMIAPAVRA 230
                               .* . :      :    .     .  *   :**::*.

Ca      SIGELFNMGFGVDVTGKIYTALRQLGFDKIFDINFGADMTIMEEATELVQRIEN------ 289
Cs      SMGELFKLGYGVDVTGKLYASMRALGFDKVFDINFGADMTIMEEATEFIERVKN------ 289
Cr      AIAETLGLAPGATTPKQLAEGLRRLGFDEVFDTLFGADLTIMEEGSELLHRLTEHLEAHP 100
So      AIGEEFGLGTGELVTGKLYGAMNQAGF-KIFDCNFAADLTIMEEGSEFIHRLHANVKGEA 263
Tm      AIQEEFGIDEDVAMAEKLVSFLKTIGFDKVFDVSFGADLVAYEEAHEFYERLKK------ 284
        :: * : :  .   . ::   :.  ** ::**  *.**:.  **. *: .*:

Ca      --NGPFPMFTSCCPGWVRQAENYYPELLNNLSSAKSPQQIFGTASKTYYPSISGLDPKNV 347
Cs      --NGPFPMFTSCCPAWVRQVENYYPEFLENLSSAKSPQQIFGAASKTYYPQISGISAKDV 347
Cr      HSDEPLPMFTSCCPGWIAMLEKSYPDLIPYVSSCKSPQMMLAAMVKSYLAEKKGIAPKDM 160
So      NAG-PLPQFTSCCPGWVRYLETRYPALLPNLSTAKSPQQMAGTVAKTYGAKVYQMQPENI 322
Tm      --GERLPQFTSCCPAWVKHAEHTYPQYLQNLSSVKSPQQALGTVIKKIYARKLGVPEEKI 342
          .  :* ******.*:   *  **  :  :*: ****   .:  *.  .    :  :.:

Ca      FTVTVMPCTSKKFEADRPQME------------KDGLRDIDAVITTRELAKMIKDAKIPF 395
Cs      FTVTIMPCTAKKFEADREEMY------------NEGIKNIDAVLTTRELAKMIKDAKINF 395
Cr      VMVSIMPCTRKQSEADRDWF---------CVDADPTLRQLDHVITTVELGNIFKERGINL 211
So      FTVSVMPCTSKKLEASRPEFNSAWQYHQEHGANSPSYQDIDAVLTTREMAQLLKLLDIDL 382
Tm      FLVSFMPCTAKKFEAEREEHEG----------------IVDIVLTTRELAQLIKMSRIDI 386
        . *:.**** *: **.*                      :* *:** *:.:::*   * :

Ca      AKLEDSEADPAMGEYSGAGAIFGATGGVMEAALRSAKDFAENAELEDIEYKQVRGLNGIK 455
Cs      ANLEDEQADPAMGEYTGAGVIFGATGGVMEAALRTAKDFVEDKDLTDIEYTQIRGLQGIK 455
Cr      AELPEGEWDNPMGVGSGAGVLFGTTGGVMEAALRTAYELFTGTPLPRLSLSEVRGMDGIK 271
So      ANTAEYQGDSLFSEYTGAGTIFGTTGGVMEAALRTAHKVLTGTEMAKLEFEPVRGLKGVK 442
Tm      NRVEPQPFDRPYGVSSQAGLGFGKAGGVFSCVLSVLNEEIG---IEKVDVKSPE--DGIR 441
         .      *   .  : **   ** :***:...*    .      :  :.    .  .*::

Ca      EAEVEINNNKYN---------------------------------------------VAV 470
Cs      EATVEIGGENYN---------------------------------------------VAV 470
Cr      ETNITMVPAPGSKFEELLKHRAAARAEAAAHGTPGPLAWDGGAGFTSEDGRGGITLRVAV 331
So      SASVSLFDTELN---------------------------------------QDVTVNVAV 463
Tm      VAEVTLKDGTSFKG--------------------------------------------AV 457
         : : :                                                    **
```

```
Ca              INGAS-NLFKFMKSGMINEKQYHFIEVMACHGGCVNGGGQPHVNPKDLEKVDIKKVRASV 529
Cs              INGAA-NLAEFMNSGKILEKNYHFIEVMACPGGCVNGGGQPHVSAKEREKVDVRTVRASV 529
Cr              ANGLG-NAKKLITKMQAGEAKYDFVEIMACPAGCVGGGGQPRSTDKA-----ITQKRQAA 385
So              VHDMGNNIEPVLRDVMAGTSPYHFIEVMNCAGGCVNGGGQP-----------IEGKGSSW 512
Tm              IYGLG-----KVKKFLEERKDVEIIEVMACNYGCVGGGGQPYPNDSR-----IREHRAKV 507
                  . .      : .        .::*:* *  ***.*****           :

Ca              LYNQDEHLSKRKSHENTALVKMYQNYFGKPGEGRAHEILHFKYKK--------------- 574
Cs              LYNQDKNLEKRKSHKNTALLNMYYDYMGAPGQGKAHELLHLKYNK--------------- 574
Cr              LYNLDEKSTLRRSHENPSIRELYDTYLGEPLGHKAHELLHTHYVAGGVEEKDEKKTSSGR 445
So              LGNI-------------------------------------------------------- 516
Tm              LRDTMGIKSLLTPVENLFLMKLYEEDLKD--EHTRHEILHTTYRPRRRYPEKDVEILPVP 565
                * :

Ca              ------------------------------------------------------------
Cs              ------------------------------------------------------------
Cr              C----------------------------------------------------------- 446
So              ------------------------------------------------------------
Tm              NGEKRTVKVCLGTSCYTKGSYEILKKLVDYVKENDMEGKIEVLGTFCVENCGASPNVIVD 625

Ca              --------------------
Cs              --------------------
Cr              --------------------
So              --------------------
Tm              DKIIGGATFEKVLEELSKNG 645
```

Figure S3. Sequence alignment of five hydrogenases Protein sequences of *Clostridium acetobutylicum* (Ca), *Clostridium saccharobutylicum* (Cs), *Chlamydomonas reinhardtii* (Cr), and *Thermotoga maritima (Tm)* HydA and *Shewanella oneidensis* HydB + HydA aligned using ClustalW web server (http://www.ebi.ac.uk/Tools/clustalw/). Catalytic site binding area highlighted in bold with critical cysteine residues in red.

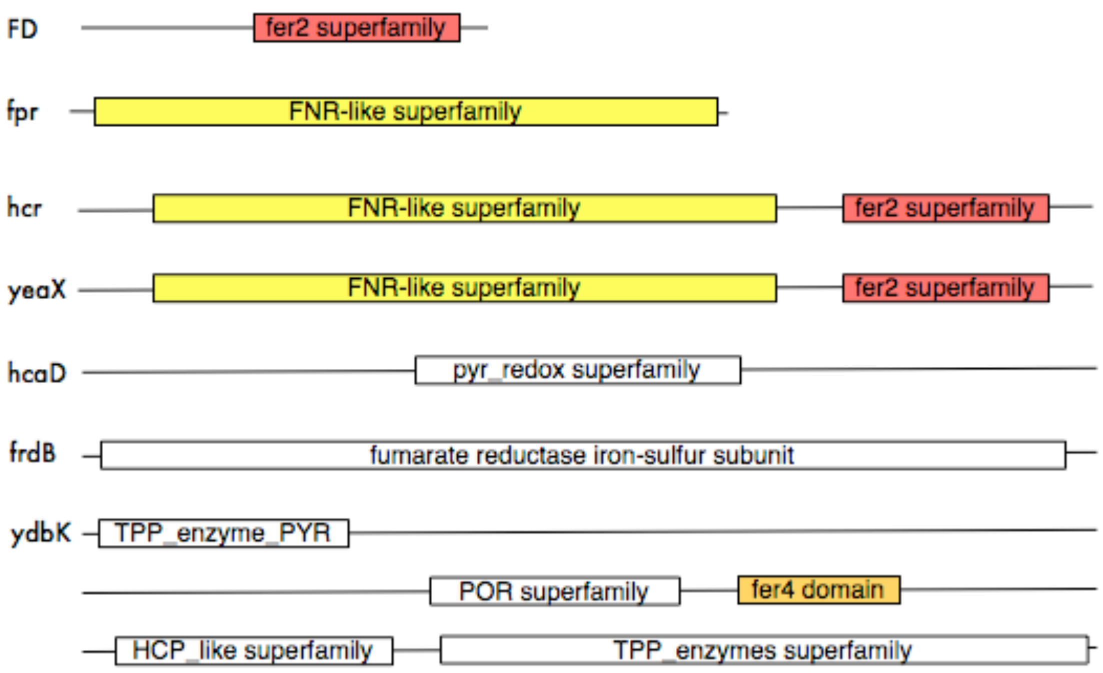

Figure S4. Deletion of competing reactions leads to hydrogen circuit insulation. A.) Domain structure of deleted ferredoxin-homology genes. FD-ferredoxin; fpr-flavodoxin:$NADP^+$ reductase; hcr-NADH oxidoreductase; yeaX-predicted oxidoreductase; hcaD-ferredoxin:NAD^+ reductase; frdB-fumarate reductase; ydbK-putative pyruvate-ferredoxin oxidoreductase. Genes were identified by BLAST homology search of the *Escherichia coli* genome against *Spinacia olearcea* ferredoxin I. Domain structure schematized from NCBI conserved domain search.

```
HydA1           APAAEAPLSHVQQALAELAKPKDDPTRKHVCVQVAPAVRVAIAETLGLAPGATTPKQLAE 60
HydA2           -ATATDAVPHWKLALEELDKPKDG-GRKVLIAQVAPAVRVAIAESFGLAPGAVSPGKLAT 58
                 .:*  .:.* : ** ** ****.  ** : .************::******.:* :**

HydA1           GLRRLGFDEVFDTLFGADLTIMEEGSELLHRLTEHLEAHPHSDEPLPMFTSCCPGWIAML 120
HydA2           GLRALGFDQVFDTLFAADLTIMEEGTELLHRLKEHLEAHPHSDEPLPMFTSCCPGWVAMM 118
                *** ****:******.*********:******.***********************:**:

HydA1           EKSYPDLIPYVSSCKSPQMMLAAMVKSYLAEKKGIAPKDMVMVSIMPCTRKQSEADRDWF 180
HydA2           EKSYPELIPFVSSCKSPQMMMGAMVKTYLSEKQGIPAKDIVMVSVMPCVRKQGEADREWF 178
                *****:***:**********:.****:**:**:**..**:****:***.***.****:**

HydA1           CVDADPTLRQLDHVITTVELGNIFKERGINLAELPEGEWDNPMGVGSGAGVLFGTTGGVM 240
HydA2           CVS-EPGVRDVDHVITTAELGNIFKERGINLPELPDSDWDQPLGLGSGAGVLFGTTGGVM 237
                **. :* :*::******.*************.***:.:**:*:*:***************

HydA1           EAALRTAYELFTGTPLPRLSLSEVRGMDGIKETNITMVPAPGSKFEELLKHR-------- 292
HydA2           EAALRTAYEIVTKEPLPRLNLSEVRGLDGIKEASVTLVPAPGSKFAELVAERLAHKVEEA 297
                *********:.*  *****.******:*****:.:*:******** **: .*

HydA1           AAARAEAAAHGTPG-PLAWDGGAGFTSEDGRGGITLRVAVANGLGNAKKLITKMQAGEAK 351
HydA2           AAAEAAAAVEGAVKPPIAYDGGQGFSTDDGKGGLKLRVAVANGLGNAKKLIGKMVSGEAK 357
                ***.* **..*:   *:*:*** **:::**:**:.**************** ** :****

HydA1           YDFVEIMACPAGCVGGGGQPRSTDKAITQKRQAALYNLDEKSTLRRSHENPSIRELYDTY 411
HydA2           YDFVEIMACPAGCVGGGGQPRSTDKQITQKRQAALYDLDERNTLRRSHENEAVNQLYKEF 417
                ************************* **********:***:.******** ::.:**. :

HydA1           LGEPLGHKAHELLHTHYVAGGVEEKDEKKTSSGRC 446
HydA2           LGEPLSHRAHELLHTHYVPGGAEADA--------- 443
                *****.*:**********.**.* .
```

Figure S5. Alignment of *Chlamydomonas reinhardtii* HydA1 and HydA2. ClustalW alignment of HydA1 and HydA2 with mutations predicted to improve the binding between HydA2 and ferredoxin (Long et. al., 2009) highlighted in red.
